# Dynamic Characteristics and Predictive Capability of Tumor Voxel Dose–Response Assessed Using ^18^F-FDG PET/CT Imaging Feedback

**DOI:** 10.3389/fonc.2022.876861

**Published:** 2022-07-06

**Authors:** Shupeng Chen, An Qin, Di Yan

**Affiliations:** ^1^ Radiation Oncology, William Beaumont Hospital, Royal Oak, MI, United States; ^2^ Radiation Oncology, Huaxi Hospital/School of Medicine, Chengdu, China

**Keywords:** FDG-PET/CT imaging feedback, tumor voxel dose–response DRM, dynamic characteristics and predictive capability, adaptive dose painting, adaptive radiotherapy

## Abstract

**Purpose:**

Tumor voxel dose–response matrix (DRM) can be quantified using feedback from serial FDG-PET/CT imaging acquired during radiotherapy. This study investigated the dynamic characteristics and the predictive capability of DRM.

**Methods:**

FDG-PET/CT images were acquired before and weekly during standard chemoradiotherapy with the treatment dose 2 Gy × 35 from 31 head and neck cancer patients. For each patient, deformable image registration was performed between the pretreatment/baseline PET/CT image and each weekly PET/CT image. Tumor voxel DRM was derived using linear regression on the logarithm of the weekly standard uptake value (SUV) ratios for each tumor voxel, such as SUV measured at a dose level normalized to the baseline SUV_0_. The dynamic characteristics were evaluated by comparing the DRM_i_ estimated using a single feedback image acquired at the *i*th treatment week (*i* = 1, 2, 3, or 4) to the DRM estimated using the last feedback image for each patient. The predictive capability of the DRM estimated using 1 or 2 feedback images was evaluated using the receiver operating characteristic test with respect to the treatment outcome of tumor local–regional control or failure.

**Results:**

The mean ± SD of tumor voxel SUV measured at the pretreatment and the 1st, 2nd, 3rd, 4th, and last treatment weeks was 6.76 ± 3.69, 5.72 ± 3.43, 3.85 ± 2.22, 3.27 ± 2.25, 2.5 ± 1.79, and 2.23 ± 1.27, respectively. The deviations between the DRM_i_ estimated using the single feedback image obtained at the *i*th week and the last feedback image were 0.86 ± 4.87, −0.06 ± 0.3, −0.09 ± 0.17, and −0.09 ± 0.12 for DRM_1_, DRM_2_, DRM_3_, and DRM_4_, respectively. The predictive capability of DRM_3_ and DRM_4_ was significant (p < 0.001). The area under the curve (AUC) was increased with the increase in treatment dose level. The DRMs constructed using the single feedback image achieved an AUC of 0.86~1. The AUC was slightly improved to 0.94~1 for the DRMs estimated using 2 feedback images.

**Conclusion:**

Tumor voxel metabolic activity measured using FDG-PET/CT fluctuated noticeably during the first 2 treatment weeks and obtained a stabilized reduction rate thereafter. Tumor voxel DRM constructed using a single FDG-PET/CT feedback image after the 2nd treatment week (>20 Gy) has a good predictive capability. The predictive capability improved continuously using a later feedback image and marginally improved when two feedback images were applied.

## Introduction

Mounting evidence has revealed that genetic and phenotypic variations exist between tumors and within each of the individual tumors (1–3). These variations result in considerable inter-tumoral and intra-tumoral heterogeneities of dose–response to radiotherapy, significantly impacting patient clinical outcomes (4–8). Therefore, targeting individual tumor heterogeneity of dose–response using a spatially non-uniform treatment dose distribution has been suggested and clinically feasible to personalize radiotherapy treatment and improve patient therapeutic ratio (9–14).

Tumor treatment response to radiation is influenced by many biological factors and changes in the tumor microenvironment (15). Most of these factors are unknown before treatment and modified dynamically during the treatment course. Tumor radiosensitivity has been estimated before treatment using *in vitro* clonogenic assay (16–18) or a linear regression model derived from the specific gene expressions (19–21). However, these methods can only measure the tumor intrinsic cellular radiosensitivity and could not be utilized to assess intra-tumoral treatment dose–response modified by tumor cell repopulation (22–24), reoxygenation (25–28), reactivation of immune response (29, 30), etc. There have been different methods to assess intra-tumoral treatment dose–response at the tumor voxel level using biological imaging feedback, i.e., acquiring PET or MR images during the treatment course (8, 14, 31–37).

Treatment feedback images have the potential to explore dynamic features of cellular activities in tumors during the treatment course, which could guide us to select the most efficient and reliable time points to quantify and estimate treatment dose–response for clinical therapeutic decisions. Most importantly, quantified intra-tumoral dose–response will guide the design of heterogeneity treatment doses to maximize the therapeutic ratio (14). In this study, serial weekly fluoro-2-deoxyglucose (FDG)-PET/CT feedback images were used to evaluate the dynamic characteristics of tumor voxel treatment response with respect to different treatment dose levels. The predictive capability of tumor voxel treatment dose–response was studied to determine the time points and minimal numbers of imaging feedback.

## Methods and Materials

### Patient Image Data and Preprocessing

The investigated patients were enrolled in an investigator-initiated clinical trial entitled “a prospective, non-randomized trial evaluating the utility of adaptive radiotherapy in the management of locally advanced head and neck squamous cell carcinomas (HNSCC) patients.” The trial was approved (IRB 2012-100) by the Hospital Review Board. In the protocol, pretreatment and weekly FDG-PET/CT imaging was planned for each patient. However, due to different clinical reasons, a number of protocol patients missed their weekly imaging partially. Thirty-one patients who had pretreatment PET/CT images and at least 3 weekly treatment PET/CT images obtained during the first 4 treatment weeks were selected for the present study. Four of 31 patients had experienced biopsy-proven local failure. The median (range) follow-up time is 23 (7~52) months. The details of the tumor characteristics are listed in **Table 1**.

**Table 1:** Tumor characteristic.

**Median age** (year)	63 (46–83)
**Gender**	
Male/female	26 (83.9%)/5 (16.1%)
**Primary site**	
Base of tongue	15 (48.4%)
Tonsil	8 (25.8%)
Supraglottic	4 (12.9%)
Unknown	2 (6.5%)
Aryepiglottic fold	1 (3.2%)
Nasopharynx	1 (3.2%)
**Clinical stage**	
II	2 (6.5%)
III	5 (16.1%)
IV	2 (6.5%)
IVA	21 (67.7%)
IVB	1 (3.2%)
**Clinical T stage**	
1	2 (6.5%)
2	17 (54.8%)
3	6 (19.4%)
4	2 (6.5%)
X	4 (12.9%)
**Clinical N stage**	
0	1 (3.2%)
1	4 (12.9%)
2a	2 (6.5%)
2b	15 (48.4%)
2c	7 (22.6%)
Unknown	1 (3.2%)
**HPV status**	
Negative/positive/unknown	6 (19.4%)/23 (74.2%)/2 (6.5%)
**Smoking**	
Non-smoker	10 (32.2%)
Light smoker (<10 pack-year)	7 (22.6%)
Heavy smoker (>10 pack-year)	14 (45.2%)

HPV, human papillomavirus.

PET/CT scans were performed on the patients 90 min after injection with 4 MBq/kg of FDG acquired in the treatment position with an immobilization mask in place using a time-of-flight Gemini TF Big Bore PET/CT scanner (Philips Medical Systems, Cleveland, OH, USA). PET images were reconstructed using the blob-ordered subsets–time-of-flight reconstruction algorithm with a voxel size of 4 × 4 × 4 mm (3). All treatments were prescribed to deliver a 2-Gy daily dose to the gross tumor volume (GTV) for 35 fractions using intensity-modulated radiation therapy or volumetric modulated arc therapy followed by the online cone-beam CT imaging-guided target position localization. Standard uptake value (SUV) of each PET voxel was calculated by normalizing the average activity concentration to the injected FDG dose per unit body weight with decay correction (38). Tumors manifested on the pretreatment PET/CT images were contoured based on a fixed SUV threshold (=2.5) and modified, if necessary, to exclude air cavities and bony structures. For a tumor that could not be delineated entirely using the cutoff SUV value due to tissue (most likely tongue) inflammation adjacent to the tumors, it was manually adjusted to the clinically used GTV boundary.

### Image Registration and Evaluation

The mean ± SD of tumor volume reduction of the 31 patients was 20% ± 18.1% at the 4th treatment week. Therefore, deformable image registration (DIR) was used to account for the tumor shrinkage in the analysis of the tumor voxel dose–response. For each patient, all weekly PET/CT feedback images were registered to the pretreatment PET/CT image using a hybrid biomechanical based DIR method (39), which includes 2 steps: 1) determine tumor boundary following an image intensity-based DIR method (ADMIRE, v1.12, Elekta Inc.) and 2) regulate the intra-tumoral mesh distribution based on finite element method.

The core of the intensity-based DIR algorithm is a local-correlation-coefficient-based dense non-linear registration algorithm with a regularization term defined as the L2 norm of the first-order spatial derivative of the displacement vector field (DVF). Previous studies have demonstrated that the intensity-based DIR achieved high accuracy for most organ boundary registration of head and neck cancer patients with the Dice similarity coefficient (DSC) ≥0.85 between the contours generated by the registration and by manual delineation (40). However, the voxel-wise displacement accuracy of image intensity-based DIR could be limited within a tumor due to the lack of distinctive image features on CT images. Therefore, the finite element method was used to correct the potential irregular displacements in tumors based on the soft-tissue mechanical characteristic. Our earlier bio-tissue phantom study has demonstrated that the uncertainty of the biomechanical-based registration, most likely, was within 4 mm (or a PET voxel size) in tumors (41). The effect of the registration uncertainty on tumor voxel dose–response assessment has been studied (42).

In this study, the tumor contours generated using the DIR on the feedback images obtained at the 2nd and 4th treatment weeks were compared to the ones manually delineated by experienced physicians. The DSC and the mean surface distance were used to evaluate the tumor boundary registration. The physical plausibility of the DVFs was evaluated using the Jacobian determinant. The Jacobian value describes the local volume change of a tumor voxel after deformation. A Jacobian value = 0.9 indicates 10% volume contraction for the tumor voxel. In comparison, 1.1 indicates a 10% volume expansion.

### Tumor Voxel Dose–Response Matrix

Tumor voxel dose–response matrix (DRM) has been quantified using tumor voxel SUV ratio manifested on the pretreatment baseline and FDG-PET/CT treatment feedback images following the DIR (14). Briefly, the logarithm of tumor voxel SUV change ratio obtained during radiotherapy was modeled using a linear random process, as follows:


(1) $$\ln\;\frac{SUV\left(v,d\right)}{SUV_{0}\left(v\right)}=A\left(v\right)\cdot d+\xi$$

where the *SUV*_0_(*v*) and *SUV*(*v*, *d*) are the pretreatment baseline SUV and the SUV after receiving a treatment dose *d* for a tumor voxel *v*, respectively. *A*(*v*) represents the average slope of the logarithm SUV change ratio during treatment up to the treatment dose *d* or the systematic component of the random process, and *ξ* is the random component representing the discrepancy between the linear model and the actual measurement at each dose level. Considering the facts of temporal variations of tumor dose–response caused by tumor reoxygenation and growth during the treatment course, slope *A* could most likely be modified by the treatment dose. However, due to the limited number of feedback images available, it has been modeled simply using the average slope. Tumor voxel DRM was quantified numerically to match the standard tumor cellular radiosensitivity index, SF_2_, to the survival fraction in 2 Gy (14), as follows:


(2) $$DRM\left(v\right)=exp\left[\frac{2}{k}\cdot A\left(v\right)\right]$$

where *k* is the calibration factor and equals 0.063 determined based on the average SF_2_ obtained from *in vitro* cellular assay of human head and neck tumors (43). DRM value represents tumor cell survival/growth in a tumor voxel during treatment; 0 < DRM < 1 implies that cell killing in the tumor voxel is larger than growth; otherwise, ≥1.

### Dose–Response Matrix Estimation

Different numbers of PET feedback images can be used to estimate the average slope *A*; thus, the DRM is based on **Eqs. 1** and **2**. Given a serial of SUVs of a tumor voxel *v* measured at different treatment dose levels, the average slope *A* can be determined using a least-squares method, as follows:


(3) $$Min{\sum_{i=1}^{N}\left[A\left(v\right)\cdot d_{i}-ln\frac{SUV\left(v,d_{i}\right)}{SUV_{0}\left(v\right)}\right]}^{2}$$

where the *SUV*(*v*, *d*_i_) is the SUV on the *i*th feedback image obtained at least 12 h after receiving a treatment dose *d*_i_, and *N* is the total number of feedback images being used. One can derive A(*v*) to be the “weighted average” of the logarithm SUV change ratios (details of derivation are in the **Supplementary Material**), as follows:


(4) $$A\left(v\right)=\left[\sum_{i=1}^{N}d_{i}\cdot ln\frac{SUV\left(v,d_{i}\right)}{SUV_{0}\left(v\right)}\right]/\sum_{i=1}^{N}d_{i}^{2}$$

The later measurement has a larger weight. In principle, the more PET feedback images are used, the more reliable the estimation should be. However, a large number of feedback images would be clinically impractical at the present time due to the extensive cost, patient inconvenience, and extra workload. In addition, an early estimation will be helpful and provide more room for treatment adaptation. Therefore, DRM estimated using 1 or 2 feedback images obtained in the early treatment should be a reasonable choice for clinical implementations and was evaluated in this study.

### Dose–Response Matrix Evaluation

Dynamic characteristics of tumor voxel dose–response were evaluated using a single PET feedback image acquired at different dose levels during the treatment course. For each patient, tumor voxel DRM_i_ was constructed using the feedback image acquired at the *i*th treatment week, *i* = 1, 2, 3, or 4. In this study, the weekly feedback images were acquired within the (mean ± SD) dose range of (7.4 ± 1.8), (17.9 ± 1.8), (26.6 ± 3.9), and (39.1 ± 3.6) Gy. The DRM_i_ was compared with the DRM_L_ constructed using the feedback image acquired in the last treatment week or within the dose range of (58 ± 9.1) Gy. DRM_L_ was used as a reference to evaluate the convergence feature of tumor voxel dose–response during treatment.

**Figure 1:**
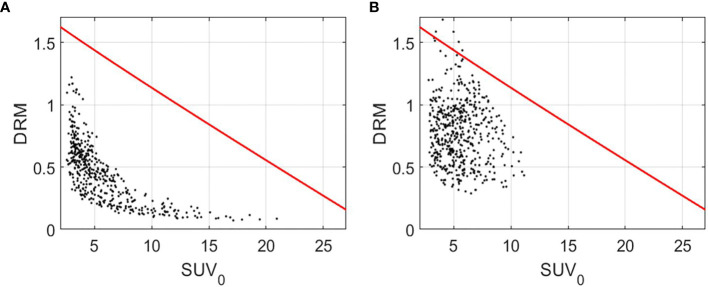
Tumor voxel (SUV_0_, DRM) for a local control tumor **(A)** and a local failure tumor **(B)** with the boundary function, $BF=-0.07\cdot SUV_{0}^{0.95}\left(v\right)+1.76$ (red curve). DRM and BF were constructed using the PET feedback image acquired during the 3rd treatment week. DRM, dose–response matrix.

The predictive capability of DRM constructed using either 1 or 2 feedback images acquired during the first 4 treatment weeks was evaluated using the receiver operating characteristic (ROC) test with respect to the treatment outcome of local tumor control or failure. As described in a previous study (14), tumor voxel control or failure is highly dependent on two factors, tumor voxel baseline SUV_0_ (tumor cell burden in the voxel) and DRM (tumor cell dose–response in the voxel). Therefore, a 2-dimensional cutoff curve or boundary function on the tumor voxel (SUV_0_, DRM) domain can be used to test the sensitivity and specificity of tumor voxel control or failure. For each estimated DRM, a boundary function, $BF=a\cdot SUV_{0}^{b}\left(v\right)+c$ , was created on the tumor voxel (SUV_0_, DRM) domain to evaluate the sensitivity and specificity of tumor voxel control or failure. The constants a, b, and c in the BF were determined by maximizing the area under the curve (AUC) for all tumor voxels. Tumor voxels above BF were those voxels that are most likely to cause tumor local recurrences. **Figure 1** shows a local control tumor and a local failure tumor with a BF superimposed on the (SUV_0_, DRM) domain. Given a treatment dose of 35 × 2 Gy, the true positive (TP) is defined such that a tumor will be locally controlled if the number of tumor voxels outside of the BF < n. The true negative (TN) is defined such that a tumor will be locally failure if the number of tumor voxels outside of the BF ≥ n. The false positive (FP) and the false negative (FN) are defined accordingly. The 95% CI of AUC was determined using the Delong method (44). The statistical significance of the AUC was determined using Mann–Whitney U-statistic (45), where the null hypothesis of AUC = 0.5. Due to the multiple tests performed in the study, the type I error rate would increase. Therefore, the conventional p-value of 0.05 for significance was adjusted to 0.002 based on the Bonferroni method (46). The sensitivity and specificity were determined by maximizing Youden’s index (47) (i.e., sensitivity + specificity − 1). Due to the imbalance of the patient dataset, F1 score = 2TP/(2TP + FP + FN) was also included in the evaluation. The predictive capability of tumor voxel DRM was compared with that of the conventional image features including maximum SUV (SUV_max_), metabolic tumor volume (MTV), and total lesion glycolysis (TLG) obtained from the pretreatment PET image and the weekly PET feedback images. The MTV was defined as the volume of the tumor voxels with SUV > 2.5. The TLG was defined as the MTV times the mean of SUV for a tumor.

## Results

**Figure 2:**
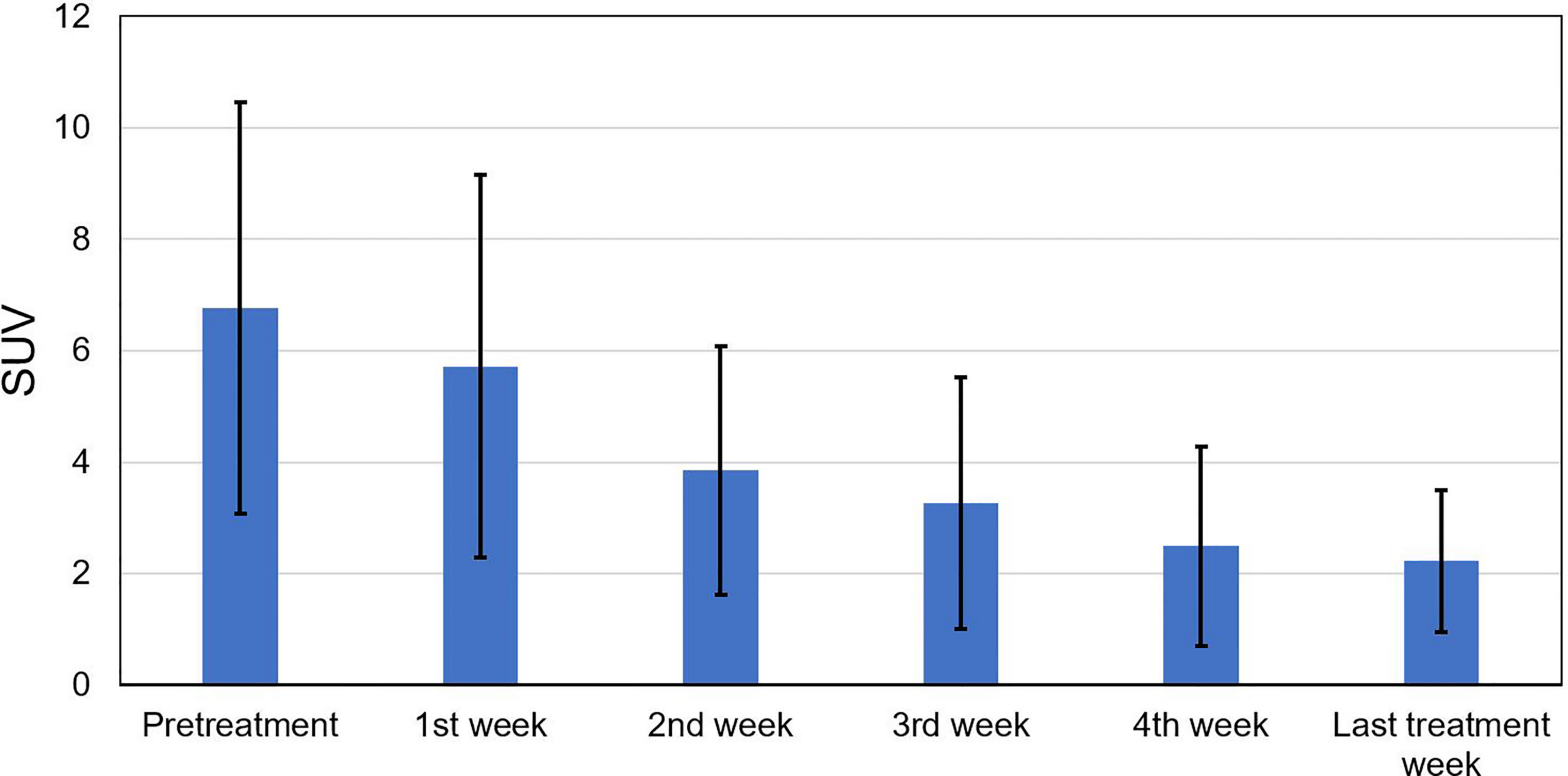
Standard uptake value (SUV) measured at the pretreatment, the 1st to 4th treatment weeks, and the last treatment week for all tumor voxels.

**Figure 2** shows the tumor voxel SUV measured during the pretreatment and different treatment weeks during radiotherapy. The SUV declined noticeably from 6.76 ± 3.69 measured at pretreatment to 3.85 ± 2.22 measured at the 2nd treatment week. After that, the SUV continuously declined and became stabilized with a mean ± SD of 3.27 ± 2.25, 2.5 ± 1.79, and 2.23 ± 1.27 for the 3rd, 4th, and last treatment weeks, respectively.

**Figure 3:**
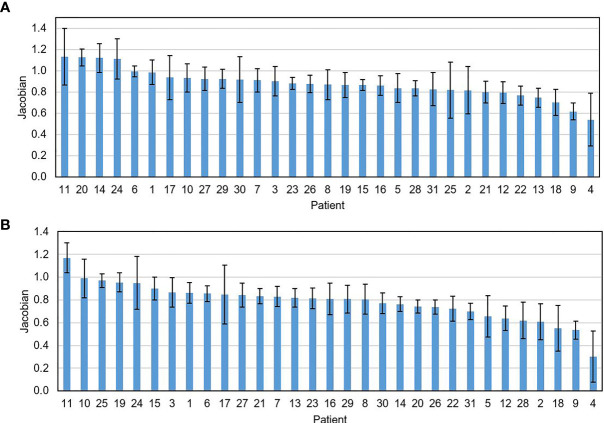
The mean ± SD of the Jacobian value for individual patients calculated on the feedback images obtained at the 2nd **(A)** and the 4th treatment weeks **(B)**.

The mean ± SD of the DSC between the contours generated by the DIR and the one manually delineated by the physicians was 0.84 ± 0.06 on the week 2 feedback image and was 0.79 ± 0.08 on the week 4 feedback image. The mean surface distances were (1.66 ± 0.54) mm and (1.85 ± 0.63) mm for the week 2 and the week 4 feedback images. No negative Jacobian value was observed for all tumor voxels. **Figure 3** shows the mean ± SD of the Jacobian values for individual patients. The Jacobian value was calculated from the DVFs obtained from the DIR performed between the pretreatment image and the week 2 and week 4 feedback images.

**Figure 4:**
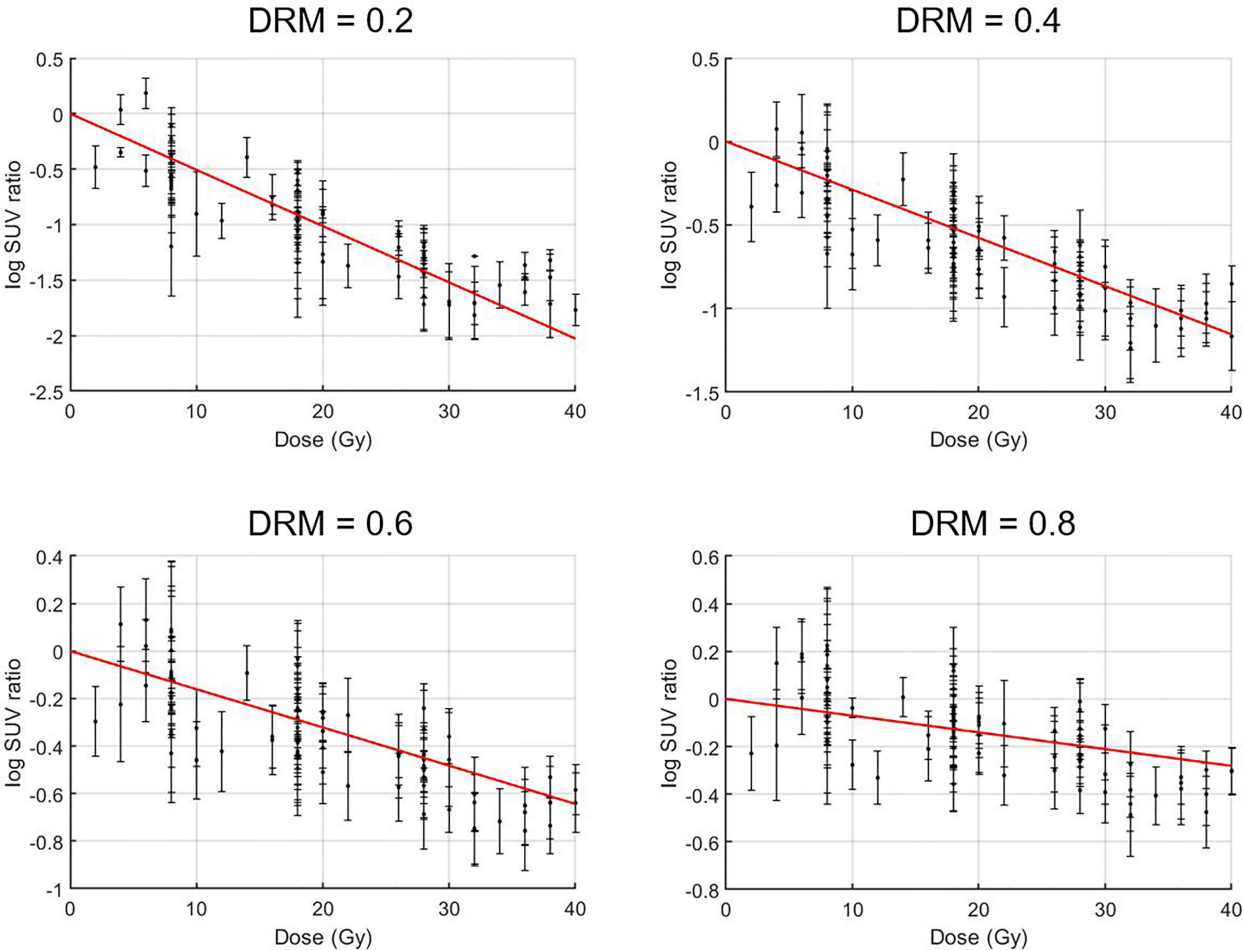
Treatment dose *versus* logarithm standard uptake value (SUV) change ratio for tumor voxels with their average dose–response DRM being 0.2, 0.4, 0.6, and 0.8. Tumor voxel SUV change ratio = SUV(*d*)/SUV_0_, i.e., tumor voxel SUV measured at a given dose level *d* normalized to its baseline SUV_0_.

A strong linear relationship between the logarithm tumor voxel SUV change ratio measured within the first 4 treatment weeks at the different treatment dose levels was identified. The mean ± SD of Pearson’s correlation coefficient was 0.91 ± 0.15 for all the 20,757 tumor voxels. As a comparison, the correlation coefficient between the tumor voxel SUV change ratio and treatment dose was lower (p-value <0.001, two-sample t-test) with the coefficient being 0.89 ± 0.18 for all tumor voxels. **Figure 4** shows the measured logarithm SUV change ratios *versus* treatment dose and the linear model (red line) from **Eq. 1** for those tumor voxels with the average dose–response DRM being 0.2, 0.4, 0.6, and 0.8, where the average DRM was determined using the first 4 weekly feedback images.

**Figure 5:**
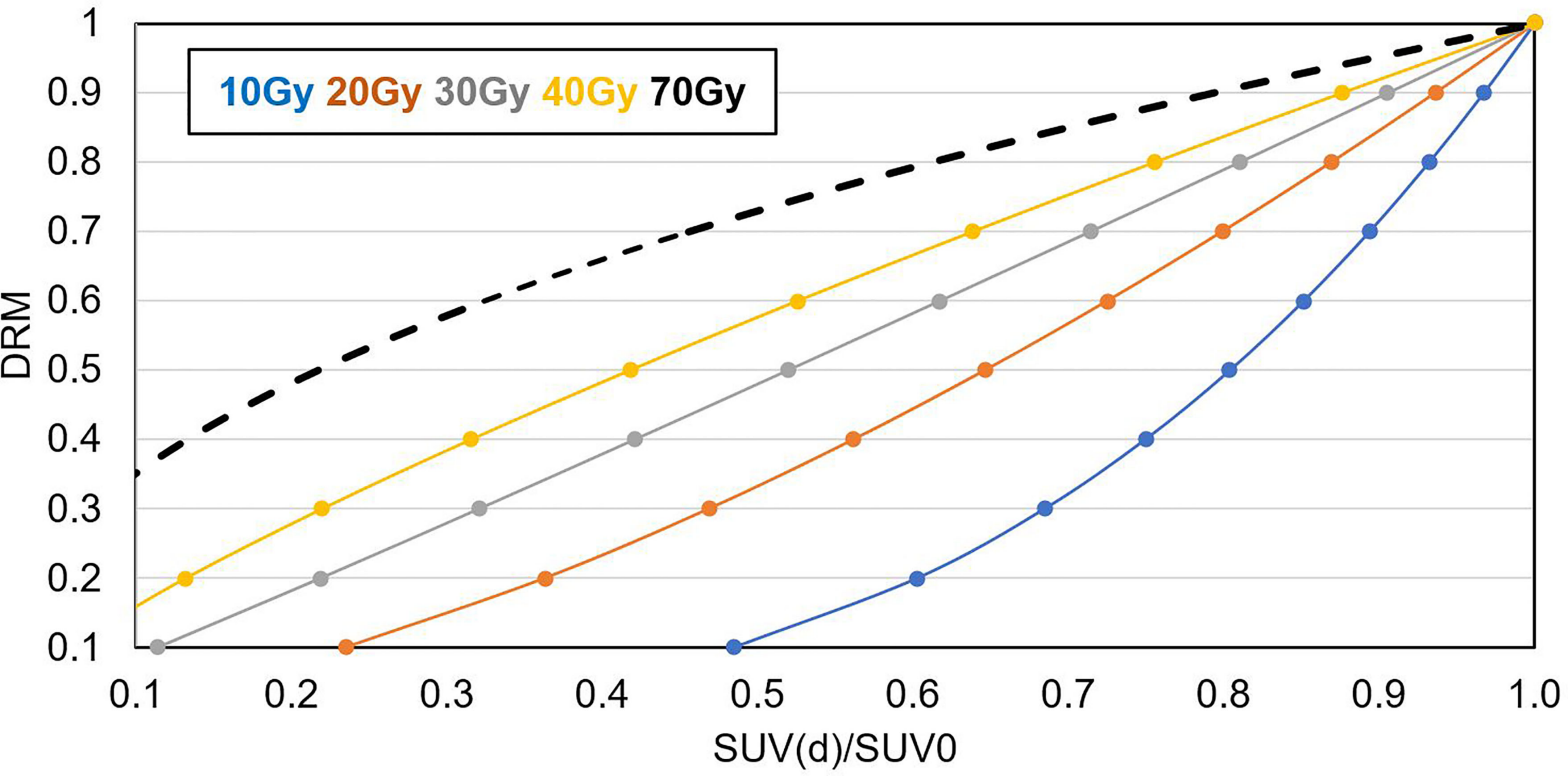
Relationship between SUV(d)/SUV_0_ and DRM for d = 10, 20, 30, 40, and 70 Gy.

**Figure 5** shows the relationship between tumor voxel DRM and tumor voxel SUV change ratio measured at different dose levels derived based on **Eqs. 1** and **2**. The slope of the curve decreased with the increase of the dose level. It implies that a DRM estimated at early treatment will be more sensitive to the errors in SUV measurement. Therefore, later DRM estimation will be more reliable.

**Figure 6:**
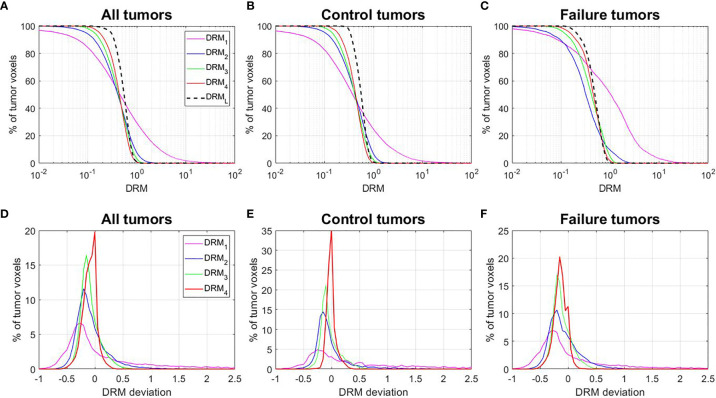
**(A–C)** Cumulative histograms of the dose–response matrix (DRM) for all tumors, control tumors, and failure tumors. **(D–F)** Histograms of the DRM deviations = DRM_i_ − DRM_L_. DRM_i_ = DRM estimated using the feedback image acquired at the *i*th treatment week; DRM_L_ = DRM estimated using the last treatment week feedback image.

**Figures 6A–C** shows the cumulative histograms of DRMs for all tumors, control tumors, and failure tumors, respectively. The distributions of DRM_i_ for both control and failure groups converge gradually to DRM_L_. Both control and failure groups have a certain number of resistant tumor voxels (e.g., DRM > 0.8), and their number gradually reduced during treatment due to reoxygenation. However, the percentage of the reduction for the failures remains smaller compared to the controls. For the controls, the percentage of tumor voxels with DRM > 0.8, V(DRM > 0.8), was 30.5%, 16%, 9.5%, and 3.6% for DRM_1_, DRM_2_, DRM_3_, and DRM_4_, respectively. For the failure patients, the corresponding V(DRM > 0.8) was 55.8%, 13.4%, 13.4%, and 7.7%. **Figures 6C–E** show the histogram of the deviation of the DRM, i.e., DRM_i_ − DRM_L_ (*i* = 1, 2, 3, and 4), for different tumor groups. For the control patients, the mean ± SD of DRM deviation was 1.22 ± 3.67, −0.01 ± 0.34, −0.04 ± 0.17, and −0.01 ± 0.08 for the DRM_1_, DRM_2_, DRM_3_, and DRM_4_, respectively. For the failure patients, the corresponding DRM deviation was 0.71 ± 4.99, −0.08 ± 0.28, −0.12 ± 0.17, and −0.14 ± 0.12, indicating the systemic underestimation of tumor voxel resistance.

**Figure 7:**
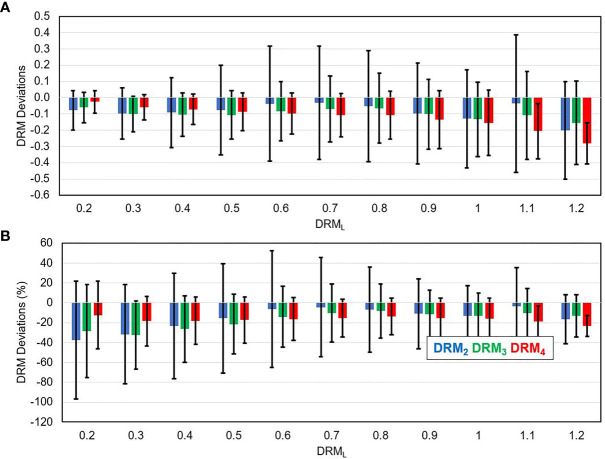
The deviation between the DRM estimated using single weekly PET feedback image (DRM_2_, DRM_3_, and DRM_4_) and the DRM_L_ estimated using the last feedback image for tumor voxels with different DRM_L_ values in absolute term **(A)** and relative term **(B)**.

**Figure 7** shows the mean ± SD of the deviation between the DRM_L_ and each of the DRMs estimated using a single feedback image for the tumor voxels within each level of DRM_L_. **Figure 7A** shows that the DRM_2_ has larger deviations as compared to the DRM_3_ and DRM_4_ for tumor voxels with respect to different levels of DRM_L_ in absolute value for all tumor voxels. In contrast, **Figure 7B** shows the relative deviations (%) decrease with the increase of the DRM_L_ level.

**Figure 8:**
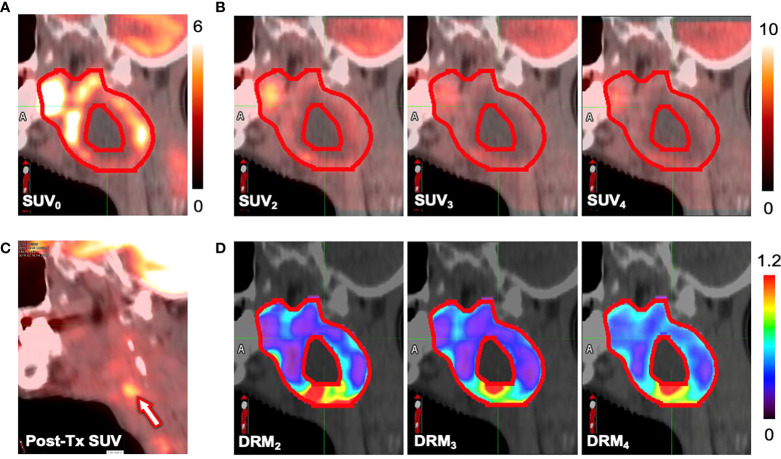
Standard uptake values (SUVs) measured at **(A)** the pretreatment (SUV_0_), **(B)** the *i*th treatment week (SUV_i_), and **(C)** the 6-month posttreatment. **(D)** The dose–response matrix (DRM) estimated using single feedback image for a patient (primary site: tonsil, stage IV, HPV−) who experienced local recurrence.

**Figures 8A, B** show the pretreatment (SUV_0_) and *i*th treatment week (SUV_i_) for a local failure tumor. **Figure 8C** shows the 6-month posttreatment (post-Tx) FDG-PET/CT image. The locally high metabolic activity region (arrow) detected the recurrence position. **Figure 8D** shows the tumor voxel DRM estimated using a single weekly feedback image. The highly resistant areas (DRM > 1) on the DRMs, predicted using different weekly PET feedback images, appeared to be consistently close to the recurrence location.

**Table 2** shows the ROC test results for the DRMs estimated using different PET feedback images. For those DRMs estimated using a single feedback image, the predictive capability quantified by AUC was improved from 0.78 for DRM_1_ to 1.00 for DRM_4_. In contrast, the predictive capability of the DRMs estimated using 2 feedback images remains high, with AUC ≥ 0.95. The predictive capability of the FDG avidity or SUV_max_ remained relatively low. The AUCs for the maximum SUV were 0.61~0.77. Both MTV and TLG achieved a moderate predictive capability with the AUC being 0.74~0.81 and 0.77~0.93, respectively. There is no clear time trend for the predictive capability of SUV_max_, MTV, and TLG. **Tables 3**–**5** summarize the details of the ROC analysis for the SUV_max_, MTV, and TLG measured at the pretreatment and different treatment weeks.

**Table 2:** ROC results for the tumor voxel dose–response matrix (DRM).

	Sensitivity	Specificity	F1 score	AUC [95 CI]	p
DRM_1_	0.78	1.00	0.88	0.86[0.67, 1.00]	0.027
DRM_2_	0.89	1.00	0.94	0.93[0.82, 1.00]	0.008
DRM_3_	0.87	1.00	0.93	0.95[0.87, 1.00]	<0.001
DRM_4_	1.00	1.00	1.00	1.00[1.00, 1.00]	<0.001
DRM_12_	0.91	1.00	0.95	0.94[0.85, 1.00]	0.021
DRM_13_	0.94	1.00	0.97	0.96[0.88, 1.00]	0.006
DRM_14_	1.00	1.00	1.00	1.00[1.00, 1.00]	0.016
DRM_23_	0.91	1.00	0.95	0.94[0.84, 1.00]	0.002
DRM_24_	1.00	1.00	1.00	1.00[1.00, 1.00]	0.003
DRM_34_	1.00	1.00	1.00	1.00[1.00, 1.00]	0.002

DRM_i_ = DRM estimated using the feedback image acquired at ith treatment week.

ROC, receiver operating characteristic; AUC, area under the curve; DRM, dose–response matrix.

**Table 3:** ROC results for the maximum SUV (SUV_max_).

SUV_max_ measured at	Sensitivity	Specificity	F1 score	AUC [95 CI]	p
Pretreatment	0.89	0.75	0.92	0.77 [0.38, 1.00]	0.047
1st week	0.91	0.67	0.93	0.72 [0.26, 1.00]	0.121
2nd week	0.52	1.00	0.68	0.72 [0.47, 0.97]	0.127
3rd week	0.32	1.00	0.48	0.61 [0.30, 0.93]	0.250
4th week	0.83	0.67	0.88	0.74 [0.37, 1.00]	0.111

ROC, receiver operating characteristic.

**Table 4:** ROC results for the metabolic tumor volume (MTV).

MTV measured at	Sensitivity	Specificity	F1 score	AUC [95 CI]	p
Pretreatment	0.59	1.00	0.74	0.81 [0.60, 1.00]	0.024
1st week	0.61	1.00	0.76	0.75 [0.51, 1.00]	0.091
2nd week	0.70	0.70	0.83	0.80 [0.58, 1.00]	0.050
3rd week	0.57	0.82	0.88	0.74 [0.43, 1.00]	0.073
4th week	0.61	1.00	0.76	0.81 [0.56, 1.00]	0.050

ROC, receiver operating characteristic.

**Table 5:** ROC results for the total lesion glycolysis (TLG).

TLG measured at	Sensitivity	Specificity	F1 score	AUC [95 CI]	p
Pretreatment	0.89	0.75	0.92	0.86 [0.64, 1.00]	0.009
1st week	0.61	1.00	0.76	0.77 [0.50, 1.00]	0.078
2nd week	0.70	1.00	0.83	0.80 [0.60, 1.00]	0.050
3rd week	0.64	1.00	0.78	0.82 [0.61, 1.00]	0.024
4th week	0.78	1.00	0.88	0.93 [0.77, 1.00]	0.008

ROC, receiver operating characteristic.

## Discussion

By utilizing tumor voxel SUV change ratio determined using serial FDG-PET/CT imaging feedback, tumor voxel dose–response can be predicted and combined with the pretreatment SUV to guide treatment dose adaptation (14). The previous study (8) has demonstrated that tumor voxel DRM assessed for head and neck squamous cell carcinoma (HNSCC) had very large intra- and inter-tumoral variations. The variation had similar numerical distribution to the variations of cellular intrinsic radiosensitivity index or *in vitro* SF_2_. In addition, the DRM index was found to strongly correlate with the expression of cancer stem cell biomarker CD44 for HNSCC patients (48). Tumor voxel DRM is a dynamic index that is constantly modified by the delivered radiation dose during treatment. Thus, DRMs, estimated using imaging feedback acquired at different treatment dose levels, could have different values reflecting the dynamic characteristic of radiation-induced tumor voxel dose–response. The current study demonstrated that the tumor voxel DRM became relatively stabilized after the 2nd treatment week (**Figure 2**) or the dose > 20 Gy (for 2 Gy per fraction treatment regimen). However, the stability was dependent on the DRM levels. For those of more resistant tumor voxels, i.e., DRM > 0.8, a larger variation could occur in the later treatment (**Figure 7**). Tumor voxel DRM estimated using either 1 or 2 feedback images acquired within the dose range of 30~40 Gy can predict tumor voxel dose–response and be used for treatment adaptation.

Tumor voxel DRM estimated using a single feedback image is most favorable in clinical practice. The estimated DRM was sensitive to the timing of the PET image feedback. The predictive capability of the DRM improved as the treatment dose level increased (**Table 2**). The feedback image acquired in the 1st treatment week or within the dose range of (7.4 ± 1.8) Gy had minimal predictive capability. The disadvantage of using a very early FDG-PET feedback imaging in tumor response assessment has been studied. A study reported that using the feedback image acquired at the 1st treatment week cannot predict histomorphological tumor response (49). Another study reported that there was an initial increase of FDG uptake in tumors after 6 Gy of radiation dose (50). These very early (dose ¾ 10 Gy) metabolic changes could be caused by the inflammatory and immune response in tumors, which obscures the changes in tumor glucose metabolism induced by therapeutic effects (50). The feedback image acquired at the 2nd treatment week had an improved predictive capability and reduced systematic deviation with respect to the DRM_L_ constructed in the latest treatment week. However, DRM estimated using the 2nd feedback image still has a larger deviation from the DRM_L_ compared to the one obtained at the 3rd or 4th treatment week (**Figure 7**). The single feedback image obtained within the 3rd or 4th treatment week was comparable with respect to the DRM_L_ (**Figure 6**). However, each of them has different advantages and disadvantages. Feedback image acquired within the 3rd treatment week will provide an early prediction and, thus, more room for clinical treatment adaptation. However, an earlier DRM estimation is more sensitive to the uncertainty in the DRM construction (**Figure 5**). Using **Eqs. 1** and **2**, one can derive the relationship between the uncertainty of tumor voxel SUV and DRM, as follows:


(5) $$\frac{\delta DRM\left(v\right)}{DRM\left(v\right)}=\frac{2}{k\cdot d}\times \frac{\delta SUV\left(v,d\right)}{SUV\left(v,d\right)}$$

Thus, the uncertainty in DRM construction caused by the SUV uncertainty is inversely proportional to the treatment dose *d*. As an example, 5% of SUV variation will cause about 7.9% of DRM variation predicted at a dose of 20 Gy, compared to 5.3% at a dose of 30 Gy. Therefore, the time point of single imaging feedback acquired after the 2nd treatment week or >20 Gy faces a tradeoff between the early clinical decision on treatment adaptation and predictive reliability. One clinical option is to select the single feedback time point based on the minimal treatment dose required in a clinical dose painting protocol.

The predictive capability of tumor voxel DRM on treatment outcome of local–regional tumor failure or control was slightly improved when using 2 PET feedback images in the DRM estimation (**Table 2**). Meanwhile, the predictive capability was less sensitive to the timing of feedback imaging. Therefore, if the clinical workload is not a major concern, using 2 feedback images acquired between the end of the 2nd and 4th treatment weeks should be favorable to be used as guidance for target dose adaptation.

Various markers have been developed to predict tumor response to radiation for HNSCC patients. A gene expression profiling created from biopsies was proved to be of high predictive value on treatment outcomes (51, 52). Specific expression patterns of microRNA have been shown to predict therapeutic response in HNSCC patients (53, 54). In addition, as shown in this study and others (55–57), the SUV_max_, MTV, and TLG obtained from a single FDG-PET image also had good predictive value on the treatment outcome. These markers are all useful in predicting treatment outcomes. However, the purpose of the study is not to demonstrate that the DRM can provide a better or equal prediction of treatment outcome than the other markers. The predictive capability quantified in the study was for evaluating the time point of image feedback for DRM construction. In fact, we used both the pretreatment SUV and DRM obtained from image feedback together to assess treatment outcome (14). Using both the pretreatment tumor voxel SUV, as a surrogate of tumor cell density in tumor voxel, and the tumor voxel DRM, as the radiosensitivity, to assess treatment outcome quantitatively also provides the spatial distribution of tumor voxel dose-efficacy. This important 3D information in tumors will be used to optimize dose distribution design for individual patients in adaptive treatment.

The major weakness of the study was that the patient cohort, especially the failure patient number, is relatively small. Therefore, the predictive capability of the patient outcome should be further validated by a larger patient cohort. To achieve a target power of 0.95, a future clinical trial will need at least 49 patients assuming the null hypothesis to be AUC = 0.5. Here, the target power was defined as the desired probability of rejecting a false null hypothesis. The patient number was estimated using a previously published method (58). Another weakness was the limited number of PET/CT feedback images for each patient used in the study. Due to different clinical reasons and because a number of patients missed 2 to 3 weekly feedback images required in the protocol, the dynamic characteristics of DRM could not be reliably explored.

## Conclusions

Tumor voxel metabolic activity measured using FDG-PET/CT feedback images fluctuated noticeably during the first 2 treatment weeks and then became stabilized thereafter. Single FDG-PET/CT imaging acquired after the 2nd treatment week or the treatment dose >20 Gy is recommended to predict tumor voxel dose–response matrix in the current clinical practice. The time point of image feedback can be selected based on clinical application; later time points should be more reliable.

## Data Availability Statement

The data that supports these findings are available on request from the corresponding author.

## Ethics Statement

The studies involving human participants were reviewed and approved by Beaumont Health Institutional Review Board. The patients/participants provided their written informed consent to participate in this study.

## Author Contributions

SC performed the data acquisition and analysis and wrote the manuscript. AQ developed the deformable image registration code and evaluated the image registration. DY designed the study, supervised the research project, and revised the manuscript. All authors listed have made a substantial, direct, and intellectual contribution to the work and approved it for publication.

## Funding

SC has been supported by Beaumont Health in this study.

## Conflict of Interest

The authors declare that the research was conducted in the absence of any commercial or financial relationships that could be construed as a potential conflict of interest.

## Publisher’s Note

All claims expressed in this article are solely those of the authors and do not necessarily represent those of their affiliated organizations, or those of the publisher, the editors and the reviewers. Any product that may be evaluated in this article, or claim that may be made by its manufacturer, is not guaranteed or endorsed by the publisher.
