# Supplementary material for: Dynamic Characteristics and Predictive Capability of Tumor Voxel Dose–Response Assessed Using 18F-FDG PET/CT Imaging Feedback
Source: Front Oncol. 2022 Jul 6;12:876861. doi: 10.3389/fonc.2022.876861 (PMC9299377; doi:10.3389/fonc.2022.876861)
Supplement: Supplementary file 1 [file Presentation_1.pdf]

Supplementary Material

Based on the least squared method, let the loss term $l(A) = \sum_{i=1}^{N} \left[A(v) \cdot d_i - ln \frac{SUV(v,d_i)}{SUV_0(v)}\right]^2$

$$= \sum_{i=1}^{N} \left\{A^2(v) \cdot {d_i}^2 - 2A(v) \cdot d_i \cdot ln \frac{SUV(v,d_i)}{SUV_0(v)} + \left[ln \frac{SUV(v,d_i)}{SUV_0(v)}\right]^2\right\}$$

Therefore, $\frac{dl}{dA} = 2 \cdot \sum_{i=1}^{N} \left\{A(v) \cdot {d_i}^2 - d_i \cdot ln \frac{SUV(v,d_i)}{SUV_0(v)}\right\}$

$$= 2 \cdot \left[A(v) \cdot \sum_{i=1}^{N} {d_i}^2 - \sum_{i=1}^{N} d_i \cdot ln \frac{SUV(v,d_i)}{SUV_0(v)}\right]$$

To minimize $l(A)$, let $\frac{dl}{dA} = 0$,

i.e., $A(v) \cdot \sum_{i=1}^{N} {d_i}^2 = \sum_{i=1}^{N} d_i \cdot ln \frac{SUV(v,d_i)}{SUV_0(v)}$

Therefore, $A(v) = \sum_{i=1}^{N} d_i \cdot ln \frac{SUV(v,d_i)}{SUV_0(v)} \ / \ \sum_{i=1}^{N} {d_i}^2$
